# Clinical Lecture on Arsenical Paralysis

**Published:** 1881-04

**Authors:** J. M. Da Costa

**Affiliations:** Pennsylvania Hospital


### CLINICAL LECTURE ON ARSENICAL PARALYSIS.

By J. M. Da Costa, M.D.
Delivered at the Pennsylvania Hospital.

*Gentlemen:*—The man whom you now see is a very different patient from the man who entered the hospital some time since. His condition has greatly changed for the better; indeed, even his features wear a different expression; so that while I am reading the clinical history you will be obliged to make a certain allowance in order to appreciate the application of the notes to the case before you. I mention this that you will understand that the comparision which you will thus make mentally between the case and the record may not suggest any exaggeration or misstatement in the record, kept since the date of his admission, September 13, 1880. While some of the features then existing are still obvious, others are at present too slight to yield any evidence in regard to the cause or form of disease from which he has suffered. The patient's name is Andrew B.; he is 19 years of age. When he entered the ward he was in such an unfavorable mental condition that it was impossible to gain from him any trustworthy history; indeed, the notes say that he was for a time completely out of his mind; but, partly from his own statements and chiefly from what could be gathered from his friends, we learned that he had had gonorrhœa, and that he also had contracted syphilis about a year before admission. This, however, had not been followed by secondary symptoms, and in the main he seems to have been a healthy person.

From his statements it also appeared that about three months before entering the hospital he began dosing himself with arsenic with a view of improving his appearance, having somewhere learned of this practice among the Styrian peasants. He commenced by taking "small pinches" of arsenic—arsenious acid, I presume—and, increasing the dose gradually, he got up to about three or four pinches in the twenty-four hours. This had been going on for three or four months before his present train of symptoms appeared. For the last ten days before he came here he had been complaining first of sore throat, and, later, of abdominal pain, vomiting, and diarrhœa; he also had headache, bleeding at the nose, some cough, and decided palpitation of the heart. Notice these symptoms. You will say, How like the beginning of typhoid fever! So they were; and, what makes the resemblance greater, on admission he was even delirious. His temperature was $102\frac{1}{2}°$ (axilla), his respiration 26, and the pulse 130 to the minute. Everything indicated a fever of low type—fever, mental wandering, dry skin, prostration. But what arrested our attention at once was what is stated very specially in the note, "his face and legs were œdematous." His tongue was coated; we also found upon examination that he had a slight right sided pneumonia and a moderate amount of erythematous eruption or roseola upon the chest and abdomen. The urine was examined the morning after admission, and found to be normal. He was kept quiet in bed, and placed on a diet consisting largely of milk; his medical treatment was an ordinary fever-mixture and effervescing draught, with a little lead and opium for his diarrhœa, which was soon controlled. He rapidly got better; but it was found on September 30, notwithstanding the evident improvement in his acute symptoms, that he could not stand upon his legs; his mind, too, was not in a healty condition.

Let us now glance over this history. Here is a man who comes into the hospital with fever, some pneumonia, diarrhœa, and in short, in many respects, the symptoms of typhoid fever. As he recovers, it is found that he cannot stand up, his legs will not support him; but it is also found that he can move his legs, although with reduced force, when he is lying down. We subsequently learn from his brother that he had an epileptic convulsion on the day before he came in. Another important point that I want you to remember is that the sensibility was good, indeed it appeared exalted; and he complained of a sense of formication, and frequently of aching pains in the muscles. He was very sensitive to tickling, and near the ankle he had some numbness—perhaps from pressure upon his heels. In making the examinations he cried and complained so much when sensation was being tested, that we were, after several efforts, obliged to content ourselves with the general result above stated. He had at that time frequent jerkings of the muscles of the legs, and powerful reflex movements were easily excited. With some reference doubtless to the syphilitic history (I was not in charge of the wards at that time), he was placed upon sirop Gilbert, which you recognize as an antisyphilitic remedy, consisting principally of iodide of potassium and biniodide of mercury. I mention these points somewhat in detail in order that you may know what the symptoms were, and what was the treatment, the better to understand the subsequent course of the case. But very little change took place under this treatment; indeed, his nutrition became worse, he was emaciated, almost idiotic in mind, rambling, incoherent, having illusions which greatly annoyed him, and made him so noisy at night that he much disturbed the other patients; he was very irritable and whining often during the day.

It was in this condition that I saw him when I first took charge of the ward. He was in a curious mental state, which I may sum up in the main as hysterical; there was great fretfulness, with frequent exhibitions of temper, alternating with spells of crying; he had to be fed like a child; he was restless, slept poorly at night, and was unable to stand; he told us that he had some pain in the legs on motion.

Now, we tested the legs, and found that the soles of the feet were very sensitive to the touch, and reflex movements increased. There was absent patellar tendon reflex, but he complained a great deal when the tendon was struck. The nails of the toes were growing, althouhg there was muscular atrophy in the thighs as well as in the legs. The elecero-muscular contractility was not lost to the faradic current; the rectus and sartorious contracted, but it required a strong current to make them contract; the sensibility to the current was poor in the legs, but better in the thighs. Ordinary tactile sensibility was not impaired; it seemed, indeed, that there was a certain degree of hyperæsthesia. Although the muscles were wasted, they did not yield to the reaction of degeneration. The atrophy was about equal on both sides, and the circumference over the largest portion of the calf was only nine inches. The arms were well developed. As you notice, his frame is rather large.

Gentlemen, I placed our patient upon very large doses of iodide of potassium, with the most marked result. He has grown fat, his appetite is good, he can feed himself now, and often asks for more ; his mind is much better ; indeed he is quite rational. His eye-ground, examined with the ophthalmoscope, shows a great deal of active congestion, a neuro-retinitis, but no choking of the disk or atrophic change in the optic nerve. He only has a slight headache occasionally. He has lost his curious peevishness and hysterical manifestations; he sleeps well at night. In every respect, mentally and physically, he has improved, and in his features markedly so.

But what about the legs? Their condition you see for yourselves. You notice a state of affairs that still exists, though to a less degree than a few months ago; they have dwindled. There has been, however, a gain of more than an inch in circumference under the treatment. Your attention is called to the curious position of the legs and feet; owing to paresis of the flexors the feet are very much extended, so as to be almost in a line with the legs. He moves the legs and kicks when requested, although feebly; you see that muscular power is still impaired, though when lying down, as he is now, there is more power than when he tries to stand, which he is still unable to do without assistance. I do not think that there was ever entire loss of muscular power, but he has certainly regained much of it, particularly within the last three or four weeks. Notice the nails, which are still growing; they have emerged now some distance and are claw-like. Notice, also, the capillary congestion of the skin around the joints, and its thin polished surface as though its nutrition were affected. Sensation in the soles of the feet is good, and a prick with a pin produces at once reflex movements: he jerks away the leg instantly. Sensation of the leg itself is good; in the thigh it is very good. Indeed, at one time, as I have already told you, he seemed to be over-sensitive to touch, but on account of his mental condition it was difficult to find out just what he felt, and I have some doubts about his sensation, as he complained very much when he was disturbed. We may conclude from our examination that still there is incomplete loss of power, without impediment of sensibility, in both lower extremities.

You saw that with an ordinary faradic current there was no contraction of the recti muscles. But when we used a very strong current the recti contracted, and so did the sartorii; that was with the slowly-interrupted current—with a perceptible interval between the shocks. With the usual current of moderate strength there was no action. Using the same strength of current in the muscles of the leg, good contraction is produced in the gastrocnemius; in the flexors, where there is the greatest loss of power, there is only the merest quiver of the muscle, even with a very strong induced current. Very much the same state of things exists in the left leg as in the right; there is scarcely any appreciable difference. But even when no movement is produced the man says that he feels the current. To complete the examination, I will add that the grasp of the right hand is deficient; the left also has a feeble grasp, but not quite so feeble as the right. The skin of the hands is soft; they are perspiring a great deal. The muscles of the upper extremities now respond to the current, although at one time they did so very feebly. They have gained in electro-muscular contractility, though never has it been lost in them to the same degree as in the legs; they have decidedly gained in power of voluntary movement, for a few months ago, you remember, he was unable to feed himself. The nails here are growing too, just as in the lower limbs. There is at present no heart or lung complication; an anæmic murmur which formerly existed has passed away; the heart is rather irritable and quick, but presents none of the signs of disease. The temperature now is normal; the urine is normal. The temperature became normal soon after the acute symptoms of the first days of admission passed away, yet until quite lately there have been occasionally, and seemingly without cause, sudden rises to 101° or 102°, lasting for a day or two—on one occasion for a week; the highest temperature reached in these irregular rises was 103.5°, on the 25th of October.

Now, what is the explanation and significance of these symptoms? Here we have one of those rare cases of marked paralysis following chronic arsenical poisoning.

As the case is so rare, I propose, first, to make out a more definite diagnosis, determining what, in my judgment, this form of paralysis here present really is; and, secondly, I shall offer some general remarks upon paralysis following arsenic.

*What form of paralysis is this?* We can say truly that it has been chiefly a spinal paralysis. I do not mean to say that there were no brain symptoms, but the form of the paralysis has been what we find especially in spinal lesions. It is, I think, a spinal palsy. Next let us consider what variety of spinal lesion exists. The parts involved in the paralysis are, as you have seen, the four extremities; the arms, as well as the legs, have been affected, though the legs have borne the brunt of the disease. We must, I think, locate the lesion high up in the cord, or we must admit that it is general and extends throughout its length. From a review of the examination, we are forced to the opinion that the disease is a diffused lesion rather than a local one. What structure of the cord has suffered the greatest injury? Here I must allude to the more minute knowledge of nerve structure and spinal disorders and some of the finer points in nerve pathology to which attention has already been comparatively recently attracted by the investigations of Erb, Charcot, Althaus, Seguin, and others. There are forms of spinal paralysis pre-eminently marked by progressive muscular atrophy, defective nutrition, and defective electro-muscular contractility—defective rather than entirely lost; indeed, it is maintained by Erb that when the muscles fail to respond to the induced current they may even act more promptly to the galvanic current and with a weaker current than in health. Paresis is marked, wasting of the limbs is marked, and nutritive changes in the skin is marked, while sensibility is preserved; and many of these symptoms, as in acute poliomyelitis, come on acutely with headache and fever, and the paralysis rapidly reaches its highest degree. The muscular wasting becomes very marked; so it is in the lateral amyotrophic sclerosis of Charcot; and the nutritive changes, in addition to the palsy, bespeak in either case, though from a different cause, a certain alteration, degenerative in character, in the ganglion cells of the anterior horns of the gray matter of the cord, which have so powerful an influence on nutrition. Now, when you look at the clinical features of the case before us, you see how closely they resemble those of a spinal paralysis with involvement of the trophic spinal centres—how in many respects they are like those of acute anterior poliomyelitis.

I think I have now proved to you what kind of pralysis it is, and what is the seat of the marked lesion of the spinal cord here produced by arsenical poisoning. You see that it is chiefly in the ganglion-cells of the anterior cornua, and, judging from the marked motor affection, we know that the disorder has also diffused itself over the anterior or antero-lateral columns. The nature of the lesion is, I think, irritative or inflammatory, attacking, very likely, connective tissue or nerve-tissue.

The case that I have been expounding to you is, I believe, an important one from the point of view that it will give us a rather more accurate insight into the nature of arsenical paralysis than we have hitherto possessed. That arsenic will produce palsies, that these may happen without marked cerebral symptoms, that in certain cases atrophy of the muscles occurs, has been long known, and you will find in medical literature a number of instances reported. But, while these establish this fact, they are mostly cases of old observers—as in the group brought together by Christison—and so placed on record as to be next to valueless from the point of view of the requirements of modern scientific diagnosis and pathology. Still, I think that any one who will take the trouble of analyzing the cases, as I have done with a number, will at least be struck with this point, which indeed I do not hesitate to formulize into a law—that arsenical poisoning, whether partial or general, is pre-eminently, I may say almost invariably, a spinal paralysis. In some instances it seems to affect a special nerve-tract at its origin, but in most instances the lesion is more diffused.

Now, as to the part of the spinal cord particularly involved, it is not always the same. The observations brought together by Jaccoud in which violent and diffuse neuralgias are mentioned forbid us to doubt that the posterior roots of the spinal nerves are sometimes implicated. But, reviewing the whole field, I have been impressed with the frequent absence of sensory phenomena; and I believe I am right in affirming that the poison exerts its influence particularly on the anterior or antero-lateral columns. I will go still further. In analyzing the cases, I have been struck with the number of instances of muscular atrophy terminating in fair recoveries; and, placing these together and examining them in the light of our modern knowledge of spinal affections, and bringing them in connection with this most striking case that we have been studying, I affirm that frequently the great ganglion-cells of the anterior cornua are involved, producing lesions similar to those of anterior poliomyelitis.

As bearing upon the subject, I will mention that in some very interesting researches in the September number of the *Archives de Physiologie* for 1875 a Russian physician, Scolosuboff, alludes to paralysis with muscular atrophy having been met with by him a number of times in the chronic arsenical poison which he states to be very common in Russia. Unfortunately, he gives no particulars and no clinical data to elucidate the matter; but, from an experimental point of view, and as aiding us in understanding the mode in which arsenic acts on the nervous system, his researches are very valuable, and may, I think, be most advantageously made use of in our argument. In animals slowly poisoned with arsenic, even where paralysis was produced, arsenic was found only in very small quantities in the muscles, while it was detected in very large quantities in the spinal cord and brain—in amounts not only much larger there than in the muscles, but also much larger than in the liver. In the most conclusive experiment cited, the proportion of arsenic was 1 in muscle to 36 5 in brain and 37 3 in spinal cord. In the most acute instances of poisoning the arsenic was first found in considerable amounts in the brain, and subsequently it rapidly accumulated in the spinal cord. The nervous symptoms of arsenic-poisoning, including the paralysis, we may, then, fairly infer, are due to the localization of the arsenic in the nervous centres. The delirum, the epileptic convulsions, the headache, of the earlier stages, bespeak plainly the brain affection; the palsy, which is generally double-sided and not an early symptom, shows the presence of arsenic and the subsequent changes in the spinal cord, and is not caused, it is evident, by direct action of the arsenic upon the muscles.

Now apply these resul s to the solution of the case before us, and see how easily most of its symptoms are explained. Here was delirium at first, great mental perturbation subsequently, a deeply-injected eye-ground: the arsenic was acting upon his brain. Indeed, there can be no doubt, if I read this clinical history correctly, that its action upon the brain preceded its action upon the extremities. He entered the hospital in this curious condition; but the resident physician informs me that he certainly was able to walk on that day, and it was not noticed until afterwards that he could not stand—arsenic acting upon the spinal cord just as in the experiments upon dogs, where the spinal cord was found in a state of congestion, with more or less inflammation. But what portion of the spinal cord is principally invaded? This—which I have already explained to you will vary according to where the arsenic chiefly accumulates and leads to secondary changes—is solved by the clinical investigation. Here marked interference with nutrition showed that the disturbance must have been chiefly in the gray matter of the anterior cornua, where are situated the large ganglion cells. Thus, applying the results of recent experimental investigation to the clinical history of the case, we have the pathological conditions unfolded, or at least made almost certain.

In concluding the review of the symptoms and the causes of the case, it may be of interest to you to know that we have used the muscle trocar of Dr. Hart upon these wasted muscles of the legs, and found that they had undergone considerable degenerative changes. Some of the fibres showed fat-globules, but the most of them had rather a waxy appearance. So far as I know, these microscopic changes have not in cases of this kind before been demonstrated. I will therefore describe them more in detail. The tissue was pale. The changes noticed in the muscular fibre were those of simple atrophy, reduction in size with faintness of striation, rather than fatty degeneration; there was also a state of things akin to waxy change, the fibres were found converted into rounded lumps, and broken up transversely, their corners rounded off, resembling the waxy degeneration sometimes found in the muscles of typhoid fever patients. They were not, however, homogeneous vitreous masses, but clouded, and granular for the most part; yet fragments were seen which were highly refractive and lustrous. There was some fatty atrophy of the connective tissues, and here and there hyperplastic changes, not only relative but absolute.

You see the remarkable improvement in the muscular system which is taking place, and you can believe me that the mind also has improved; so that we are now able to assume that the disorder of the spinal cord, like that of the brain, will soon pass away; at least it will do so to a very great extent.

It only remains to speak of the treatment, and what remedy has been especially serviceable. Iodide of potassium in large doses, in my opinion, has been the principal agent in effecting the beneficial change. Iodide of potassium is not only very effective in eliminating mineral salts from the system, but at the same time it favors better nutrition after morbid processes. It is used in various forms of metallic poisoning, and some of its good effects in this case I attribute to its power of elimination. But I think that it has acted this part alone; it has also been a great modifier of tissue-change, and especially of pathological change. However this may be, we cannot doubt the result; there has been great improvement. You could hardly believe the patient to be the same person if you had seen him, as we did, a few months ago, so emaciated and almost idiotic. He has been taking twenty grains of the potassium salt three times a day, which comparatively large dose he has borne well and fattened under it. Previous to its administration, while mainly on general tonics, he was not gaining. He has good diet, and to the affected muscles the faradic current is applied every second day.

You may ask, Will complete recovery take place here? In the brain, yes. I do not believe that there will be any alteration or serious impairment of the mental powers; the brain cells will be restored to their normal functions. Will he recover fully the use of his limbs? In the upper extremities, yes; but in the lower extremities I scarcely think that he will ever do so entirely. He may walk about, even without crutches, but I hardly believe that he will recover the use of these wasted flexor muscles of the foot.

Judging by the improvement of the last month, he will come very near to a recovery; but he will never be entirely a healthy man as he was before he began the habit of taking arsenic in pinches for its beautifying effect.

March 7.—I add this note as to his present state. His general condition is good; he has continued to gain in flesh, weighs 132 pounds. He can now read with the aid of the same glasses that he used before he fell ill; has no headache, only occasional vertigo; the muscular system is well developed in the upper half of the body; the legs are gaining in size—left calf measures 11½ inches, right, 11¼ inches. He can walk with a cane, and go up and down stairs by himself. The appetite and digestion are good. The skin is in good condition except in the feet, where there is some erythema and scaling; around the toe-nails there is superficial ulceration. He sleeps well at night, and is in good spirits.—*Philadelphia Medical Times.*

